# Involvement of role of HMGB1-NLRP3 pathway in systemic disorders

**DOI:** 10.3389/fcell.2025.1600596

**Published:** 2025-07-04

**Authors:** Lei Yang, Xue Li, Xiaoming Zhu, Futao Ge, Yuantao Wang

**Affiliations:** ^1^ Department of Urology, The First Hospital of Jilin University, Changchun, China; ^2^ Department of Cardiology, Affiliated Hospital of Changchun University of Traditional Chinese Medicine, Changchun, China; ^3^ Department of Pulmonology, Affiliated Hospital of Changchun University of Traditional Chinese Medicine, Changchun, China; ^4^ Lithotriptic Center, The First Hospital of Jilin University, Changchun, China

**Keywords:** HMGB1, NLRP3, inflammation, systemic disorders, therapeutic targets

## Abstract

High mobility group box-1 (HMGB1) is a protein released from stressed or damaged cells that triggers immune activation and chronic inflammation. The NOD-like receptor (NLR) family pyrin domain-containing 3 (NLRP3) is a central component of the inflammasome, which activates caspase-1 and releases pro-inflammatory cytokines, including IL-1β and IL-18. The HMGB1/NLRP3 axis plays a critical role in regulating inflammation and immune responses, driving systemic inflammation and disease progression. Targeting this pathway offers promising therapeutic strategies for conditions such as autoimmune disorders, trauma, and chronic inflammatory diseases. In particular, inhibiting HMGB1 or NLRP3 can mitigate the exaggerated inflammatory response, reduce tissue damage, and slow disease progression. This review explores the bidirectional interactions between HMGB1 and NLRP3 and discusses current and emerging therapeutic approaches targeting this axis to modulate inflammation and improve clinical outcomes.

## 1 Introduction

The high mobility group box-1 (HMGB1) protein, a non-histone nuclear protein widely expressed in eukaryotic cells, plays a central role in DNA modulation and transcriptional regulation. Over the past few decades, HMGB1 has gained increasing recognition for its pivotal involvement in inflammation and the pathogenesis of systemic diseases (Goodwin et al., 1973; Rauvala et al., 2007; Chi et al., 2015). Upon cellular stress or injury, HMGB1 is released, either actively or passively, as a damage-associated molecular pattern (DAMP). It interacts with receptors such as toll-like receptors (TLRs) and the receptor for advanced glycation end products (RAGE), amplifying signaling pathways that drive inflammatory and immune responses (Yang et al., 2015; Harris et al., 2012). The NOD-like receptor (NLR) family pyrin domain containing 3 (NLRP3) inflammasome is another key player in inflammatory diseases. Composed of the sensor protein NLRP3, the adaptor protein ASC, and pro-caspase-1, it recognizes cellular damage and infections, triggering the release of pro-inflammatory cytokines like IL-1β and IL-18, and inducing pyroptosis (Bauernfeind et al., 2009; Yang et al., 2019). The relationship between HMGB1 and NLRP3 forms a critical feedback loop: HMGB1 activates NLRP3 through NF-κB signaling and ROS/ATP pathways, while NLRP3 activation further promotes the release of HMGB1, exacerbating inflammation (Yang et al., 2020a). This bidirectional interaction sustains chronic inflammation, contributing to the progression of various systemic diseases.

The HMGB1/NLRP3 axis plays a critical role in mediating the transition from localized inflammation to systemic responses, acting as a molecular bridge in chronic inflammation and immune dysregulation. Understanding the role of the HMGB1/NLRP3 signaling pathway is especially pertinent for systemic diseases such as autoimmune disorders, cardiovascular conditions, and neurodegenerative diseases. Inflammation driven by the HMGB1/NLRP3 axis contributes to disease progression and tissue damage in these contexts. This review provides a comprehensive analysis of the mechanistic roles of HMGB1 and NLRP3 in systemic inflammation and explores their potential as therapeutic targets for systemic diseases.

## 2 Structure and function of HMGB1 and NLRP3

### 2.1 HMGB1

**FIGURE 1:**
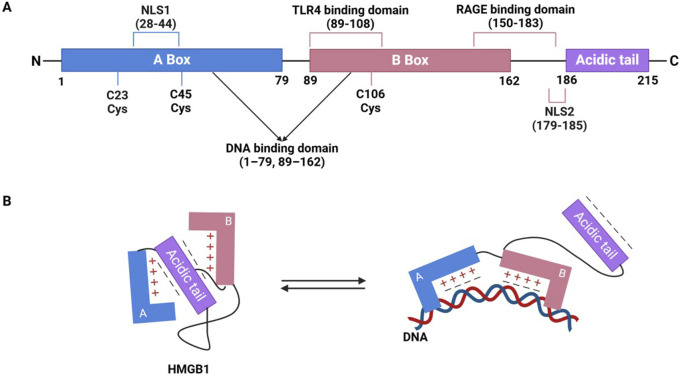
The structure of HMGB1 and its interaction with DNA. **(A)** Domain and functional sites of HMGB1. **(B)** Conformational transition of HMGB1 and its DNA binding mode.

HMGB1 is a multifunctional protein that plays a central role in regulating inflammation and immune responses. It consists of 215 amino acids and has a molecular weight of approximately 30 kDa. Its structure features two highly conserved DNA-binding domains, known as the A box (amino acids 1–79) and B box (amino acids 89–162), along with a C-terminal acidic tail (amino acids 186–215) (Figure 1A). These domains are arranged in an “L” shape, facilitating their interaction with DNA. The A and B boxes are positively charged, enabling them to bind to the minor groove of DNA, thereby inducing DNA bending and subsequently altering nucleosome stability and chromatin conformation (Figure 1B). The B box functions as the primary inflammatory region, while the A box acts as its antagonistic counterpart. The functions of these domains are influenced by the redox state of three key cysteines (Cys23, Cys45, and Cys106) (Kang et al., 2014; Frank et al., 2015a; Wang et al., 2019). The C-terminal acidic tail of HMGB1, rich in glutamic and aspartic acids, carries a negative charge and plays a role in its functions, while a short N-terminal region of lysine residues contributes to its interactions and biological activity (Chen et al., 2022; Bianchi et al., 1992). HMGB1 contains two nuclear localization signals (NLS1 and NLS2) that mediate its nuclear translocation. Structurally, the protein contains specific binding domains that facilitate interactions with pattern recognition receptors (PRRs), particularly TLR and RAGE, thereby promoting its diverse biological functions through these receptor-mediated pathways (Taverna et al., 2022) (Figure 1A).

Normally, HMGB1 is located in the nucleus, where it acts as a non-histone chromatin protein. By binding to DNA, it regulates chromatin structure and influences key processes such as gene expression, DNA repair, and replication (Bianchi and Agresti, 2005). However, under stress conditions such as infection, injury, or inflammation, HMGB1 can be released into the extracellular space, where it acts as DAMP. This release can occur through intrinsic immune activation or passive release following cellular damage, involving various cell types like immune cells, fibroblasts, and epithelial cells (Chen et al., 2022; Pisetsky, 2014; Bonaldi et al., 2003). Once outside the cell, HMGB1 binds to various receptors, thereby activating immune cells, amplifying inflammatory responses, and driving disease progression (Rapoport et al., 2020). Post-translational modifications, such as acetylation, phosphorylation, and oxidation, regulate its shuttling between the nucleus and cytoplasm, influencing processes like apoptosis, autophagy, and cell death, which ultimately determine (Tang et al., 2007; Wu et al., 2013; Ge et al., 2014; Cui et al., 2019; Kang et al., 2011).

The functional activity of HMGB1 is predominantly modulated by its redox state. In its unoxidized (reduced) form, HMGB1 primarily functions as a chemokine, recruiting immune cells to sites of injury or inflammation without triggering a significant inflammatory response. However, when oxidized, HMGB1 exhibits pro-inflammatory properties, activating signaling pathways such as NF-κB and MAPK, which amplify the inflammatory response (Park et al., 2003; Dumitriu et al., 2005). In addition, evidence indicates that redox status alone does not fully explain HMGB1’s pleiotropic functions. Its extracellular activity is also critically shaped by the type, expression level, and activation state of cell surface receptors within the local microenvironment. These receptors mainly include classical PRRs such as TLR4 and RAGE, as well as non-canonical receptors like TIM-3 (Tang and Lotze, 2012) and CXCR4 (Pirani et al., 2024), which are differentially expressed across immune cell subsets and pathological states, thereby contributing to distinct immunological outcomes in processes such as inflammation, tumor progression, and tissue repair. This dual regulation by redox state and receptor context adds a layer of complexity to HMGB1’s immunomodulatory functions. At lower concentrations, HMGB1 contributes to antimicrobial activity and transient inflammation. However, during infection, necrosis, or apoptosis, HMGB1 levels rise significantly, leading to severe inflammation, epithelial barrier disruption, organ dysfunction, and even death. Active HMGB1 also plays a role in various pathological conditions, including fever, anorexia, acute-phase reactions, and vascular leakage syndrome (Andersson and Tracey, 2011).

### 2.2 NLRP3

**FIGURE 2:**
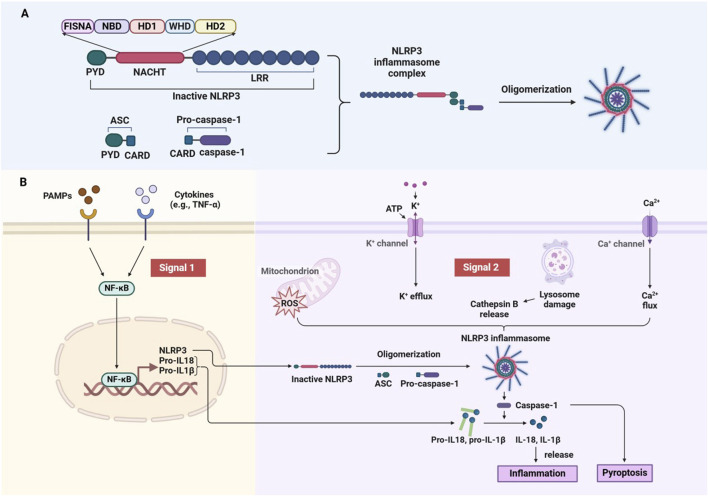
Structural domains and activation mechanism of the NLRP3 inflammasome. **(A)** Domain of NLRP3 and assembly of the Inflammasome. **(B)** Two-Signal model for NLRP3 inflammasome activation.

As a key sensor of the intrinsic immune system, NLRP3 sensitively detects endogenous cellular damage and exogenous pathogenic invasion. Structurally, NLRP3 contains three main functional domains: the Pyrin domain (PYD) for protein stabilization, the leucine-rich repeat (LRR) domain for protein-protein interactions, and the NACHT domain, responsible for nucleotide binding and ATP hydrolysis (Sharif et al., 2019; Swanson et al., 2019). In addition, the NACHT domain includes several subdomains: the Fish-specific NACHT-associated domain (FISNA), Nucleotide-binding domain (NBD), helical domains (HD1, HD2), and a winged helical domain (WHD), essential for NLRP3’s function (Fu and Wu, 2023; Zhang et al., 2015; Dekker et al., 2021) (Figure 2A).

NLRP3 activation occurs in response to a wide range of endogenous and exogenous stimuli, including ATP, microbial agonists, particulate matter, and pore-forming toxins. The activation process is triggered by two signals. The initial signal is provided by PAMPs or cytokines (e.g., TNF-α), which activate the NF-κB pathway to induce NLRP3 and pro-IL-1β expression. The second signal is triggered by microbial or danger signals, leading to K^+^ efflux (Muñoz-Planillo et al., 2013), Ca^2+^ influx (Lee et al., 2012), cathepsin B leakage from lysosomes (Hornung et al., 2008), ROS production (Zhou et al., 2010), translocation to mitochondria (Zhou et al., 2011), and mitochondrial dysfunction (Iyer et al., 2013). When activated, NLRP3 forms a complex with apoptosis-associated speck-like protein containing a caspase recruitment domain (ASC) and caspase-1 (Agost et al., 2004). This complex drives the maturation and release of inflammatory cytokines such as IL-1β and IL-18, which promote further immune activation and recruit additional immune cells to the site of injury or infection (Schroder and Tschopp, 2010). Additionally, NLRP3 activation can lead to a form of programmed cell death called pyroptosis, which exacerbates inflammation and tissue damage (Boucher et al., 2018) (Figure 2B). While essential for pathogen defense, dysregulated NLRP3 activity is associated with various inflammatory and autoimmune diseases, such as arthritis, cardiovascular disease, and neurodegenerative disorders. Therefore, understanding how NLRP3 is activated and regulated is crucial for developing therapies aimed at modulating its activity in disease contexts.

## 3 Inflammatory mechanisms of the HMGB1/NLRP3 axis

**FIGURE 3:**
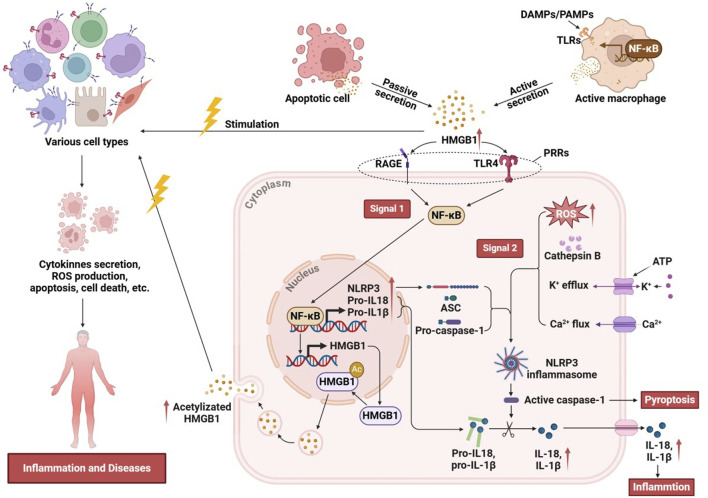
The inflammatory mechanisms of the HMGB1/NLRP3 axis.

The interaction between HMGB1 and the NLRP3 inflammasome has emerged as a critical regulator of inflammatory responses across various pathological contexts. HMGB1, acting as a DAMP, interacts with immune receptors to initiate and amplify inflammation, particularly by engaging the NLRP3 pathway. The HMGB1/NLRP3 axis orchestrates a series of pro-inflammatory events that drive acute and chronic inflammatory states, making it a central focus in understanding the mechanisms of immune activation, tissue injury, and disease progression. HMGB1 can be actively secreted by cells exposed to inflammatory mediators, DAMPs, or PAMPs, or it can be transiently and passively released from apoptotic cells or from monocytes activated by exposure to apoptotic cells (Andersson and Tracey, 2011; Gauley and Pisetsky, 2009; Qin et al., 2006). Extracellularly secreted HMGB1 functions as a pro-inflammatory mediator, eliciting rapid responses from various cell types, including monocytes and macrophages (Li et al., 2003), dendritic cells (Yang et al., 2007), neutrophils (Fan et al., 2007), T-cells (Dumitriu et al., 2005), B-cells (Tian et al., 2007), epithelial cells (Yang, 2006), and smooth muscle cells (Porto et al., 2006) and so on. Extracellular HMGB1 binds to PRRs, through signaling pathways such as NF-κB, and activates NLRP3 inflammasomes, thereby delivering the “signal 1” required for NLRP3 activation (Wang et al., 2018; Vogel et al., 2018; Lamkanfi and Dixit, 2014). The relationship between HMGB1 and NLRP3 is not only that the former upregulates NLRP3 expression. In addition, HMGB1 may, under specific pathological conditions, enhance intracellular stress responses, such as ROS production, K^+^ efflux, and mitochondrial dysfunction, to facilitate the delivery of signal 2 required for NLRP3 inflammasome activation. Although these events are well-established canonical activation pathways, HMGB1 can act in concert with other stimuli to amplify and modulate the inflammasome activation process (Jiao et al., 2021; Lu et al., 2012). Once the NLRP3 inflammasome is assembled, caspase-1 is activated to cleave pro-IL-1β and pro-IL-18, generating their active forms, IL-1β and IL-18. Activation of the HMGB1/NLRP3 axis enhances the secretion of these proinflammatory cytokines, thereby amplifying both local and systemic inflammatory responses (Wang et al., 2020). Activated caspase-1 induces cellular pyroptosis, during which HMGB1 is further released as a DAMP that diffuses into surrounding tissues and activates the NLRP3 inflammasome in neighboring cells (Figure 3). Pyroptosis is also accompanied by the release of other DAMPs, amplifying the positive feedback loop of the HMGB1/NLRP3 axis and perpetuating a vicious cycle of inflammation (Wang et al., 2020). In summary, the HMGB1/NLRP3 axis plays a central role in amplifying both local and systemic inflammatory responses by activating the inflammasome and driving the secretion of pro-inflammatory cytokines. This pathway, through its dual-signal mechanism involving ROS generation and ionic flux alterations, mediates acute inflammation and contributes to chronic inflammatory states observed in various pathologies. Elucidating the intricacies of the HMGB1/NLRP3 axis underscores its potential as a therapeutic target for controlling inflammation-related diseases and enhancing immune regulation.

## 4 Role of HMGB1/NLRP3 axis in systemic diseases

### 4.1 Nervous system

#### 4.1.1 Neuroinflammation

Neuroinflammation is a critical mediator in the initiation and progression of various brain disorders. Microglia, the resident immune cells of the central nervous system, are essential for sensing pathological insults and maintaining neural homeostasis. However, dysregulated microglial activity can lead to sustained inflammation, thereby accelerating neurodegenerative processes (Kwon and Koh, 2020; Niraula et al., 2017). For instance, Liu et al. demonstrated that exposure to PM2.5 particles in the atmosphere activates mouse microglia and induces neuroinflammation through the HMGB1/NLRP3 signaling pathway. Silencing HMGB1 in this context downregulated the NLRP3 inflammasome and the subsequent NF-κB/MAPK pathways, reduced pyroptotic cell death, mitigated hippocampal neuron damage, and improved synaptic function in neurons (Liu et al., 2022a). Similarly, Liao et al. found that inhibition of the HMGB1/NLRP3 axis in a rat stroke model reduced pro-inflammatory factors (IL-6, IL-1β, and TNF-α) in the brain, promoted the release of anti-inflammatory factors (IL-10, TGF-β, and brain-derived neurotrophic factors), and facilitated the shift of microglia from the M1 to M2 phenotype. This resulted in the preservation of blood-brain barrier integrity and improved the prognosis of ischemic stroke-related brain injury (Liao et al., 2024). Furthermore, Ye et al. (2019) emphasized the pivotal role of the TLR4/NF-κB pathway in mediating the interaction between HMGB1 and NLRP3 during microglial polarization, thus further driving neuroinflammation. As previously discussed, the redox state of HMGB1 significantly impacts its activity. This was corroborated by Frank et al. (2015b), who demonstrated that the disulfide form of HMGB1 exhibited pro-inflammatory effects both *in vivo* and *in vitro*. This form of HMGB1 directly induced microglial polarization toward an immune phenotype via TLR4 signaling, with NLRP3 playing a pivotal role in the downstream neuroinflammatory responses. Prolonged exposure to such inflammatory stimuli may further exacerbate the neuroinflammatory process.

#### 4.1.2 Depression

Depression is a leading mental health disorder, with increasing global prevalence (Monroe and Harkness, 2022). Inflammatory pathways, including the HMGB1/NLRP3 axis, contribute to the pathophysiology of depression. Studies suggest that targeting this axis may offer therapeutic benefits. In depressed rats, overactivation of the hypothalamic-pituitary axis and elevated levels of HMGB1, RAGE, and NLRP3 were observed in the hippocampus. HMGB1 amplifies inflammation by activating RAGE and TLR4, activating the NF-κB signaling pathway, leading to the synthesis and release of pro-inflammatory cytokines. Additionally, caspase-1-specific inhibitor ameliorated depressive behaviors by inhibiting the pyroptosis of oligodendrocytes in the hippocampus during depression (Yang et al., 2020b). Recent studies have demonstrated that downregulation of HMGB1 and NLRP3 protein expression has been linked to alleviating depressive symptoms, making the HMGB1/NLRP3 axis a potential target for antidepressant therapies (Xie et al., 2021; Hendawy et al., 2023). These studies provide compelling evidence for the involvement of HMGB1 and the NLRP3 inflammasome in depression-associated neuroinflammatory pathways, where elevated activation levels correlate with both acute and chronic depression. By amplifying inflammation, the HMGB1/NLRP3 axis contributes to both the onset and persistence of depression, positioning it as a potential therapeutic target for inflammation-related depression subtypes.

#### 4.1.3 Brain injury and cognitive dysfunction

Traumatic brain injury (TBI) is closely associated with cognitive dysfunction and neuroinflammation, with activation of the HMGB1/NLRP3 pathway playing a key role in these processes. Following TBI, increased Tau protein levels are observed in the dentate gyrus and thalamus, which correspond with elevated HMGB1 and NLRP3 activation, contributing to cognitive deficits. Inhibition of this axis has been found to improve spatial short-term memory, hippocampal spatial working memory, and restore nesting activity in mice (Zhao et al., 2021a). Moreover, a study by Shan et al. demonstrated that prenatal exposure to sevoflurane impairs learning and memory in rat offspring through HMGB1-induced activation of NLRP3/ASC inflammasome (Shan et al., 2023). Notably, the HMGB1/NLRP3 axis is also implicated in cognitive dysfunction associated with sepsis (Xiong et al., 2022; Ma et al., 2024), diabetes (Liu et al., 2022b), and Alzheimer’s disease (Elariny et al., 2024). Targeting this axis may attenuate these pathologies, though further clinical validation is needed. Additionally, the neuronal NLRP3 inflammasome serves as critical mediator of cortical neuroinflammation and trigeminal vascular activation. Microglia activation may further promote neuronal NLRP3 inflammasome-mediated cortical neuroinflammation through the HMGB1–TLR2/4 pathway, thereby contributing to migraine development (Chen et al., 2023a). In a study of 48 COVID-19 patients with headache symptoms, those with headaches exhibited a stronger inflammatory response compared to those without headaches. Elevated levels of HMGB1 and NLRP3 were implicated in the induction of the trigeminal system, with NLRP3 levels showed a moderate positive correlation with headache severity, length of hospitalization, and headache duration, while HMGB1 levels were positively correlated with headache severity (Bolay et al., 2021).

Ischemic brain injury, a critical factor affecting prognosis, further underscores the role of the HMGB1/NLRP3 axis. In a study by Zhang et al. (2024a), neutral polysaccharide from *Gastrodia elata* was able to attenuate the expression levels of HMGB1 and NLRP3 after cerebral ischemia/reperfusion injury. This alleviated neuroinflammation mediated by ferroptosis through the NRF2/HO-1 signaling pathway, although the precise relationship between anti-ferroptosis and anti-inflammatory effects remains unclear. Both Stress and cerebral ischemia synergistically increase HMGB1 levels in the serum and hippocampus, with activated microglia being the main source of its release. This activation further triggers the NLRP3 inflammasome pathway, impairing autophagic function in microglia and aggravating injury (Espinosa-Garcia et al., 2020). Notably, HMGB1 is released from cells during cerebral hemorrhage and binds to TLR4 on the surface of immune cells. Activation of the NLRP3 inflammasome promotes pyroptosis following cerebral hemorrhage (Lei et al., 2024). A case-control study investigating the correlation between NLRP3 expression and HMGB1 levels in the serum of children with febrile epilepsy revealed that serum levels of HMGB1, NLRP3, caspase-1, IL-1β, IL-6, and TNF-α were significantly higher in children with epilepsy compared to controls (Ye et al., 2024). Although they did not further explore how HMGB1 and NLRP3 interact to influence epileptogenesis, it focused on clinical cases and provided a valuable direction for future research. Additionally, the HMGB1/NLRP3 axis plays a significant role in both acute and chronic inflammation, contributing to neurological tumorigenesis and progression. In gliomas, HMGB1 has been shown to promote M1-like polarization of macrophages activate the NF-κB pathway by binding to RAGE, and enhance the release of the NLRP3 inflammasome. Bioinformatics analysis further revealed that HMGB1 levels were higher in glioma tissues than in non-tumor tissues, and elevated intracellular HMGB1 expression correlated with poor prognosis (Li et al., 2022).

#### 4.1.4 Retinal damage and glaucoma

The HMGB1/NLRP3 axis is crucial in the progression of ocular diseases, particularly in retinal damage and glaucoma. For instance, infection with *Staphylococcus pseudintermedius* activates the NLRP3/HMGB1/ROS/GSDMD signaling axis in corneal epithelial cells, exacerbating apoptosis and leading to the formation of characteristic focal holes, numerous extracellular bubbles, and other localized phenomena (Wang et al., 2024a; Wang et al., 2024b). The increased release of HMGB1 regulates NLRP3 activation through an NF-κB-dependent mechanism, contributing to retinal damage and potentially progressing to glaucoma. This poses a serious threat to human vision and may result in irreversible blindness. Interestingly, inhibition of the HMGB1/NLRP3 axis has been shown to reduce inflammation and mitigate retinal injury, offering a potential strategy to protect vision and prevent irreversible blindness (Chi et al., 2015; Zhao et al., 2024a).

#### 4.1.5 Other neurological disorders

Spinal cord injury (SCI) is a significant public health issue that imposes a substantial burden on both patients and society. Primary SCI causes cell death and vascular destruction, which subsequently trigger secondary processes, including inflammation, ischemia, and oxidative stress, that exacerbate the injury. The activation of inflammasomes plays a critical role in driving the tissue inflammatory process (Alizadeh et al., 2019). Recent research have shown that activating autophagy can inhibit the HMGB1/NF-κB/NLRP3 pathway, thereby improving motor function and reducing tissue damage caused by inflammatory responses following SCI (Gholaminejhad et al., 2022). Müller et al. demonstrated that lipocalin 2 increased the expression of HMGB1 and NLRP3 in SCI, and knocking down lipocalin 2 reduced gliosis and NLRP3 activation in astrocytes (Müller et al., 2023). Heatstroke, which causes vascular endothelial injury and multi-organ damage, also involves HMGB1 in the activation of NLRP3 inflammasomes. Regulating the HMGB1/NLRP3 axis could mitigate heatstroke-induced damage, suggesting a potential therapeutic strategy for these conditions (Zhang et al., 2024b; Pei et al., 2023). Additionally, Yin et al. (2022) delved into the thrombocytopenia that occurs after heatstroke from another direction, and they found that after heatstroke, HMGB1 induces high levels of ROS production via TLR4 and RAGE, which further activates NLRP3 inflammasomes involved in platelet activation and reduction *in vivo*.

### 4.2 Respiratory system

#### 4.2.1 Acute lung injury (ALI)

Acute lung injury (ALI) involves damage to lung tissue, leading to dysfunction of alveoli, capillaries, and bronchioles. This inflammatory syndrome, which can progress to acute respiratory distress syndrome, is marked by the involvement of various inflammatory factors (Burkard et al., 2023; Matthay and Zemans, 2011). The HMGB1/NLRP3 axis plays a central role in this process. Activation of NLRP3 in ALI models induces expression of triggering receptor expressed on myeloid cells-1, which activates ROS–NF-κB signaling via HMGB1 and IL-18 secretion, creating a positive feedback loop that amplifies the inflammatory response (Zhong et al., 2020). Furthermore, inhibition of the HMGB1/NLRP3/Caspase-1 or HMGB1/RAGE/NF-κB signaling pathways in the lung attenuates inflammatory infiltration and cellular pyroptosis, thereby reducing lung injury (Ding et al., 2023; Chen et al., 2021; Wu et al., 2024). Targeting the expression and activation of these pathways may represent a novel strategy for ALI.

#### 4.2.2 Pulmonary fibrosis (PF)

If left untreated, ALI can progress to pulmonary fibrosis (PF). Huang’s team found that bleomycin-induced acute lung injury in rats led to patchy fibrosis, atelectasis, hyperinflation, thickened alveolar septa, and significant inflammatory cell infiltration in lung tissues, accompanied by increased NLRP3 expression. Inhibition of HMGB1 expression promoted the nuclear translocation of nuclear factor erythroid 2-related factor 2 (NRF2) and enhanced HO-1 levels, thereby reducing NLRP3 expression and partially reversing lung fibrosis (Huang et al., 2023). The NLRP3 inflammasome has also been shown to regulate epithelial-mesenchymal transition in the development of pulmonary fibrosis, while HMGB1 promotes fibroblast proliferation and collagen accumulation. Furthermore, inhibition of the HMGB1/TLR4 axis and NLRP3 inflammasome/NF-κB signaling pathways attenuates both inflammation and fibrosis (Aydin et al., 2023; Elkhoely et al., 2023; Yang et al., 2024a). Targeting these pathways may offer new therapeutic options for PF, though clinical validation is needed.

#### 4.2.3 Asthma

Asthma is a heterogeneous chronic inflammatory disease of the respiratory system that causes airflow obstruction and affects approximately 10% of adults. Among these, up to 6% have severe asthma, which is associated with a reduced quality of life and an increased risk of exacerbations, hospitalization, and death (Settipane et al., 2019; Varricchi et al., 2022). The HMGB1/NLRP3 axis plays a pivotal role in asthma initiation and progression. HMGB1 activates the NF-κB signaling pathway through TLR4 binding, which subsequently activates and releases the NLRP3 inflammasome. Additionally, HMGB1 induces autophagy in bronchial epithelial cells, further promoting NLRP3 inflammasome activation (Meng et al., 2023). Similarly, pre-treatment with ROS scavengers and NF-κB inhibitors in human bronchial epithelial cells downregulates NLRP3 inflammasome proteins, reduces IL-1β and IL-18 levels, and improves mitochondrial membrane potential (Jiao et al., 2021). Li et al. (2018) found that, in a mouse model of ovalbumin-induced asthma, the ATP/P2X7 axis activated NLRP3 inflammasomes, inducing the expression and release of HMGB1 in dendritic cells. HMGB1 was shown to regulate allergic airway inflammation, mucus production, and Th2 and Th17 polarization in asthmatic mice.

#### 4.2.4 Other respiratory conditions

Inflammation and immunity play critical roles in the pathogenesis of pulmonary hypertension, with HMGB1 acting as a key DAMP released by ferroptotic cells to activate the NLRP3 inflammasome via binding to TLR4. Inhibition of ferroptosis in pulmonary artery endothelial cells attenuates pulmonary vascular remodeling and protects the right ventricle in pulmonary hypertensive rats (Xie et al., 2022). Additionally, HMGB1’s interaction with RAGE and NF-κB exacerbates inflammation through a positive feedback loop in the lung tissues of patients with drug-associated pulmonary toxicity (Abd Elmaaboud et al., 2024). Notably, although hyperactivation of the HMGB1/NLRP3 axis often plays a detrimental role in humans, Gao and Huang (2024) observed that is oflurane treatment in lung cancer cells activated HMGB1 and RAGE, upregulated NLRP3 expression, and promoted pyroptosis in tumor cells without affecting the viability of normal human bronchial epithelial cells. This finding suggests that isoflurane may be a more suitable anesthetic option for lung cancer surgery patients.

### 4.3 Digestive system

#### 4.3.1 Liver injury

The importance of HMGB1 and the NLRP3 inflammasome in liver diseases has garnered increasing attention. In acute liver injury, macrophages release extracellular vesicles containing HMGB1, which are taken up by hepatocytes through transferrin-mediated endocytosis via binding to RAGE or TLR4. This process activates the NLRP3 inflammasome, triggering hepatocyte pyroptosis and injury. These findings suggest that HMGB1 not only serves as a marker of liver cell injury but also actively participates in regulating inflammatory responses (Wang et al., 2021; Chen et al., 2024a). Autophagy is essential for maintaining liver homeostasis. He et al. found that inducing autophagic flux reduced HMGB1 secretion in hepatocytes, which subsequently inhibited macrophage NLRP3 inflammasome activation, offering a potential therapeutic strategy for liver fibrosis (He et al., 2023). Conversely, autophagy-deficient hepatocytes actively release HMGB1, promoting cholangiocyte proliferation and tumorigenesis. This is driven by increased transcription of Caspase-11, triggered by sustained NRF2 activation, which activates inflammasomes and enhances HMGB1 release (Khambu et al., 2023). In alcoholic liver disease, HMGB1 translocates from the nucleus to the cytoplasm in hepatocytes, increasing apoptosis signal-regulating kinase 1-positive hepatocytes, passive HMGB1 release, and NLRP3 inflammasome activation, promoting inflammation and hepatic fibrosis (Adjei-Mosi et al., 2023). Inhibiting the NLRP3/HMGB1 signaling pathway reduces the feedback loop between inflammation and oxidative stress, protecting hepatocytes (Cao et al., 2022; Yao et al., 2024). Studies on hepatic ischemia-reperfusion injury (IRI) have demonstrated that hepatic IRI upregulates the expression of inflammation-related proteins, including HMGB1, TLR4, and NLRP3, which triggers inflammatory responses and hepatocyte injury (Du et al., 2021; Tong et al., 2023; El-Sisi et al., 2021). Xie et al. (2020) found that HBV protein X induced an increase in mitochondrial ROS under oxidative stress, activating the NLRP3 inflammasome. This activation led to hepatocyte death and the release of inflammatory factors, including IL-1β, IL-18, and HMGB1. Furthermore, the expression of inflammasome components, such as NLRP3 and IL-1β, was positively correlated with the HBV DNA load, suggesting the involvement of the NLRP3 inflammasome pathway in the progression of HBV-related hepatitis.

#### 4.3.2 Gastrointestinal disorders

The release of HMGB1 is closely linked to the development of various diseases, including acute pancreatitis and gastrointestinal injury (Wu et al., 2021; Arab et al., 2022). In acute pancreatitis, HMGB1 upregulation promotes the activation of the NLRP3 inflammasome, leading to the release of pro-inflammatory cytokines, including IL-1β, which further exacerbates pancreatic injury (Wu et al., 2021). HMGB1 plays a critical role in ethanol-induced gastric ulcers by binding to TLR4 and RAGE, activating NF-κB, and inducing the activation of the NLRP3 inflammasome, which delays ulcer healing. Conversely, inhibiting HMGB1 expression accelerates the healing process (Alzokaky et al., 2020). In bile reflux gastritis, pyroptosis mediated by the NLRP3 inflammasome results in the release of HMGB1, which contributes to the inflammatory response. Additionally, ubiquitin-specific protease 50 interacts with ASC to deubiquitinate and activate the NLRP3 inflammasome, promoting the release of HMGB1. This release subsequently drives gastric tumorigenesis through the PI3K/AKT and MAPK/ERK pathways, revealing a novel mechanism in bile reflux-associated gastric carcinogenesis (Zhao et al., 2024b). Activation of the NLRP3/HMGB1 pathway disrupts intestinal tight junction proteins and promotes intestinal epithelial cell death, thereby compromising the integrity of the intestinal barrier (Li et al., 2024). Ulcerative colitis is a chronic inflammatory bowel disease affecting the colon and rectum, significantly impairing patients’ quality of life and causing long-term, burdensome complications (Le Berre et al., 2023). Bioinformatics analysis revealed high expression levels of several inflammation-associated genes, including TNF-α, HMGB1, and NLRP3, in the colonic tissues of patients with ulcerative colitis (Gao et al., 2024). Experiments have demonstrated that the anti-inflammatory effects achieved by inhibiting HMGB1 binding to TLR4 and suppressing the activation of the NF-κB/NLRP3 inflammasome pathway can ameliorate colonic mucosal barrier disruption and neutrophil recruitment in ulcerative colitis (Chen et al., 2023b; Sun et al., 2024). A prospective observational study revealed elevated serum levels of NLRP3 and HMGB1 in patients with ulcerative colitis, which were positively correlated with disease severity (Chen et al., 2020). Although the sample size of this study was small, it provides clinical evidence supporting the role of NLRP3 and HMGB1 in ulcerative colitis and suggests their potential as novel diagnostic markers for the disease.

### 4.4 Urinary system

In acute kidney injury, HMGB1 mediates cellular pyroptosis and renal tissue injury by binding to and activating TLR4 and RAGE receptors, triggering the NF-κB signaling pathway, and promoting downstream activation of NLRP3 and proinflammatory mediators (Hassan et al., 2024; Yang et al., 2024b; Henedak et al., 2024). Inhibition of HMGB1/NF-κB/NLRP3 pathway-related protein expression in HK-2 cells exerts anti-oxidative stress and anti-apoptotic effects. Treatment with glycyrrhizin analogs to inhibit HMGB1 levels accelerated the excretion of urea nitrogen and serum creatinine in septic mice, attenuated renal tissue injury, and preserved the integrity of the brush border (Qiang et al., 2023). Zhang et al. (2023) showed that DAMP accumulated after ferroptosis in renal cells recruits macrophages, activates HMGB1/RAGE/TLR4/MyD88 signaling, activates the NF - κB signaling pathway, and induces NLRP3 accumulation and cellular pyroptosis. Hypertension and diabetes mellitus, two major contributors to the global burden of chronic diseases, often lead to renal fibrosis during the progression of chronic kidney disease. HMGB1 and the NLRP3 inflammasome serve as key mediators of renal fibrosis and may even contribute to renal failure in hypertensive nephropathy and diabetic nephropathy (Awa et al., 2024; Zhang et al., 2020).

### 4.5 Genital system

Recurrent spontaneous abortion (URSA) is a common pregnancy complication with a complex etiology. In approximately 50% of cases, the underlying mechanism remains unknown. However, inflammation and abnormal immune tolerance are thought to be important pathogenic factors. Zhu et al. (2021) demonstrated that HMGB1, GSDMD, NLRP3, and caspase-1 proteins were elevated in the decidua tissues of URSA mice and patients. These results suggest that HMGB1, actively secreted by macrophages, may induce cellular pyroptosis via activation of the NF-κB signaling pathway, leading to aseptic inflammation, disruption of the maternal-fetal interface, and ultimately triggering URSA. Research indicates that HMGB1 and NLRP3 also contribute to pro-inflammatory processes in endometriosis and pre-eclampsia (Nunes et al., 2022; Huang et al., 2022). The HMGB1/RAGE/NLRP3 axis plays a pivotal role in cervical epithelial pyroptosis, promoting tumor cell proliferation and inflammation (You et al., 2021).

### 4.6 Circulatory system

#### 4.6.1 Cardiac injury

Studies have shown that HMGB1 is upregulated in macrophages after cardiac injury and contributes to myocardial inflammation by promoting NLRP3 inflammasome activation under hypoxic conditions (Hu et al., 2022). Fan et al. found that elevated plasma levels of NLRP3 and HMGB1 were associated with poor prognosis in congenital heart disease, suggesting their potential as prognostic biomarkers for the condition (Fan et al., 2020). Furthermore, the HMGB1/NLRP3/caspase-1 pathway plays a role in hypoxia-reoxygenation-induced cellular pyroptosis in cardiomyocytes. Targeted inhibition of HMGB1 levels mitigates cytotoxicity and reduces the release of inflammatory factors (Fei et al., 2022). Song et al. demonstrated that Lipocalin-2 induces NLRP3 inflammasome activation through the HMGB1/TLR4 signaling pathway in mice with pressure overload-induced cardiac dysfunction, thereby impairing cardiac function (Song et al., 2017). Inhibiting HMGB1-dependent NLRP3 inflammasome activation reduces inflammation and apoptosis, enhances cellular antioxidant defenses, and shows potential for treating septic myocardial dysfunction and myocardial infarction (Zhao et al., 2021b; Wei et al., 2024).

#### 4.6.2 Hematological disorders

Acute myeloid leukemia (AML) is the most common acute leukemia in adults and can be either *de novo* or secondary to other processes (Bhansali et al., 2023). Using a mouse model of AML, Liu et al. (2021a) found that chronic restraint stress caused weight loss and reduced survival in AML mice. The proposed mechanism suggests that stress-induced HMGB1 secretion promotes AML progression by activating NLRP3 inflammasomes. The upregulation of the platelet NLRP3 inflammasome in patients with sickle cell disease is dependent on HMGB1/TLR4. This upregulation may regulate platelet responses and contribute to platelet aggregation, thrombosis, and vascular leakage through an autocrine or paracrine IL-1 receptor-mediated feed-forward loop (Vogel et al., 2018). Inhibiting the HMGB1/NLRP3 signaling pathway could serve as a potential therapeutic target for treating inflammation-induced thrombosis and vascular remodeling diseases in the future (Wei et al., 2022; Kim et al., 2018; Zhang et al., 2024c). During Burkitt lymphoma development, lysate cells express elevated levels of HMGB1, which activate the NLRP3 inflammasome to sustain ZEBRA expression. ZEBRA initiates the transition of the virus from the latent to the lytic state in lysate cells, thereby maintaining the lytic signal. This process facilitates viral replication and dissemination within host cells and may contribute to lymphoma progression (Reinhart et al., 2022). In sepsis, lipopolysaccharide, a cell wall component of Gram-negative bacteria, stimulates macrophages, leading to the release of HMGB1, an important inflammatory mediator. HMGB1 provides an inflammatory microenvironment conducive to the activation of NLRP3 inflammasomes. In turn, inflammatory factors released by activated NLRP3 inflammasomes further amplify HMGB1 release and inflammatory signaling. Together, these processes escalate the inflammatory response in sepsis, resulting in severe tissue injury, organ dysfunction, and ultimately poor patient prognosis (Xie et al., 2016). Studies have shown that the expression levels of NLRP3 and HMGB1 are markedly elevated in patients with myelodysplastic syndromes and myelofibrosis, suggesting their potential involvement in disease pathogenesis (Basiorka et al., 2016; Apodaca-Chávez et al., 2022; De Luca et al., 2024; Parciante et al., 2023). Although the direct mechanistic linkage between the two molecules remains to be fully elucidated, their concurrent upregulation under analogous pathological conditions suggests that the HMGB1/NLRP3 axis may play a pivotal role in driving disease progression and could serve as a promising target for therapeutic intervention.

### 4.7 Motor system

Following tendon injury, macrophages undergo cellular pyroptosis, and HMGB1 translocates from the nucleus to the cytoplasm, where it is subsequently packaged into extracellular vesicles and released into the extracellular space. The HMGB1/TLR axis activates the NF-κB signaling pathway in tendon-derived stem cells, working synergistically with NLRP3 inflammasomes to induce tendon-derived stem cell senescence and aberrant osteogenic differentiation, ultimately contributing to traumatic ectopic ossification (Li et al., 2023). HMGB1 triggers the assembly and activation of the NLRP3 inflammasome, leading to extracellular matrix disorganization, inflammation, and impaired healing after tendon injury (Thankam et al., 2018). Macrophages play a crucial role in maintaining tissue homeostasis and immune regulation, with M1-polarized macrophages promoting the production of inflammatory factors and influencing disc degeneration. While attenuating M1-polarized macrophage-mediated nucleus pulposus cell injury through inhibition of the HMGB1-MyD88-NF-κB pathway and NLRP3 inflammasome (Zhao et al., 2021c). The levels of HMGB1, NF-κB, NLRP3, IL-1β, and TNF-α proteins were significantly elevated in bone marrow mesenchymal stem cells following SCI, while inhibiting the HMGB1/NF-κB/NLRP3 inflammatory pathway, attenuating spinal cord tissue destruction, and improving the motor function of rats with SCI (Gholaminejhad et al., 2022).

### 4.8 Immune system

**FIGURE 4:**
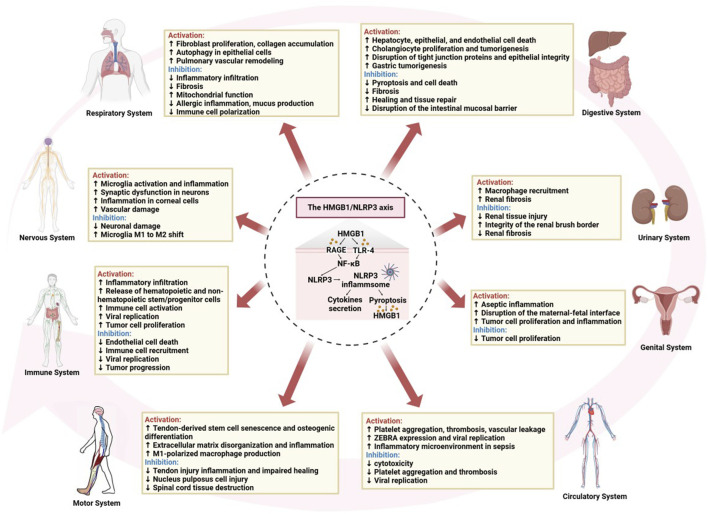
The roles of activation or inhibition of the HMGB1/NLRP3 axis in systemic diseases.

Macrophage exposure to inflammasome agonists induces the autophosphorylation of double-stranded RNA-dependent protein kinase, which broadly regulates inflammasome activation through physical interactions with various inflammasome components, including NLRP3, and is critical for caspase-1 activation, IL-1β cleavage, and HMGB1 release (Lu et al., 2012; Yu et al., 2019). HMGB1 also plays a crucial role in aseptic inflammation by activating the NLRP3 inflammasome, inducing IL-1β secretion and sepsis, and recruiting neutrophils and eosinophils, resulting in inflammatory infiltration and tissue damage (Jessop and Holian, 2015; Noguchi et al., 2021). Activation of the HMGB1/MyD88/NF-κB/NLRP3 signaling pathway plays a central role in inflammation in endothelial cells, which are classical antigen-presenting cells (Liu et al., 2021b; Yu et al., 2022). Interestingly, diurnal changes in ATP levels in peripheral blood regulate the diurnal release of hematopoietic and non-hematopoietic stem/progenitor cells from the bone marrow to peripheral blood through the activation of NLRP3/HMGB1 (Adamiak et al., 2020). In atopic dermatitis, HMGB1 activates the secretion of the NLRP3 inflammasome and inflammatory factors in epidermal keratinocytes by interacting with the RAGE receptor, as well as with IFN-γ and TNF-α, either individually or synergistically, leading to erythema and inflammation in the skin (Chang et al., 2023). Binsaleh et al. (2024) identified a strong association with the skin inflammatory response, influencing disease severity and patient depression, suggesting a potential link between this axis in skin inflammation and associated psychological states. Kawasaki disease is an immune-mediated vasculitis in which the body’s immune system is abnormally activated, with immune cells releasing large amounts of inflammatory mediators, resulting in vascular endothelial cell damage and an inflammatory response (McCrindle et al., 2017). Studies have shown that endothelial cellular pyroptosis is a key pathophysiological event in Kawasaki disease, triggered by high levels of HMGB1, leading to elevated RAGE expression and increased histone B activity, ultimately resulting in NLRP3 inflammasome-dependent, caspase-1-mediated endothelial cellular pyroptotic death. Circulating HMGB1 may serve as a sensitive predictor of endothelial injury in Kawasaki disease, and the inhibition of pyroptosis activation may represent a potential therapeutic target (Jia et al., 2019). Moreover, in addition to promoting viral replication and the inflammatory response during viral infection (Zhang et al., 2024d), HMGB1/NLRP3 is also involved in various biological processes in tumor cells, including cell proliferation, apoptosis, and immune regulation, thus offering new targets and strategies for tumor therapy (Chen et al., 2024b; Yang et al., 2024c) (Figure 4).

## 5 Potential therapeutic targets of the HMGB1/NLRP3 axis

Given the central role of the HMGB1/NLRP3 axis in various systemic diseases, therapeutic strategies targeting HMGB1 and NLRP3 have progressively emerged as a prominent area of research. By modulating the activation of this axis, it is possible to reduce the inflammatory response, block inflammation-induced tissue damage, and improve disease outcomes. Current therapeutic strategies focus on two primary approaches: first, inhibiting the release of HMGB1 or blocking its interaction with TLR/RAGE, and second, targeting NLRP3 to prevent inflammasome activation and subsequent pro-inflammatory cytokine release.

Recent advancements have highlighted a range of promising therapeutic agents targeting HMGB1 and NLRP3. Anti-HMGB1 monoclonal antibodies have shown efficacy in preclinical models of inflammatory diseases, such as sepsis and acute liver injury, by neutralizing HMGB1 and reducing the inflammatory response. Recombinant HMGB1 box A proteins, which specifically bind to the receptor-binding domain of HMGB1, have been shown to attenuate tissue damage in models of traumatic injury and chronic inflammation (Yang et al., 2020a; Andersson et al., 2018; Wang et al., 1999). Additionally, endogenous molecules such as immunoglobulins, antithrombin, thrombomodulin, vasoactive intestinal peptide, and growth hormone-releasing peptide have demonstrated potential in reducing HMGB1-mediated inflammation in various conditions, including infections and chemical toxicity (Yang et al., 2020a; Lu et al., 2014). Among natural compounds, niacin and mung bean extracts have been found to effectively inhibit HMGB1 release, offering novel therapeutic options for inflammatory diseases. Exogenous herbal ingredients are particularly promising due to their low toxicity and broad therapeutic potential, but their clinical development still faces hurdles, such as standardization of dosages and efficacy validation (Lu et al., 2014). In the field of NLRP3 inhibition, numerous compounds are currently under extensive investigation. MCC950, a potent small-molecule inhibitor of NLRP3, has demonstrated significant efficacy in attenuating inflammatory responses across both preclinical studies and early-phase clinical trials, showing therapeutic potential for a range of diseases, including Alzheimer’s disease, type 2 diabetes mellitus, and cardiovascular disorders (Li et al., 2022). Andrographolide, a bioactive natural compound isolated from Andrographis paniculata, has also been reported to effectively suppress NLRP3 inflammasome activation and ameliorate inflammatory pathologies in conditions such as asthma and rheumatoid arthritis (Agrawal and Nair, 2022). Additionally, synthetic small molecules, such as JC121, which exhibit high selectivity for NLRP3 targeting, have emerged as promising candidates for the treatment of inflammasome-mediated diseases (Blevins et al., 2022). Recent research efforts have facilitated the development of clinically promising NLRP3-targeted compounds. Dapansutrile Klück et al. (2020) and Inzomelid (NCT04015076), as next-generation NLRP3 inhibitors, have successfully entered clinical trials and demonstrated favorable safety profiles. These advances represent a critical step forward in the clinical translation of NLRP3-targeted therapies. HMGB1 and the NLRP3 inflammasome engage in a reciprocal activation process, establishing a positive feedback loop that amplifies inflammatory responses. Inhibiting either HMGB1 or the NLRP3 inflammasome alone reduces the activation of the other to some extent, attenuating the pro-inflammatory effects of the HMGB1/NLRP3 axis.

While these therapeutic strategies have shown potential, challenges remain in translating preclinical findings into clinical success. One of the primary concerns is the selectivity of inhibitors. Many compounds targeting NLRP3 also affect other immune signaling pathways, which can lead to unintended side effects. HMGB1 and NLRP3 are critically involved in multiple immune regulatory and inflammatory processes, maintaining a delicate and dynamic balance between inflammatory homeostasis and pathological responses. Although pharmacological inhibition of these key mediators holds promising therapeutic potential, it may also lead to immune dysregulation, aberrant inflammatory responses, and even tumor progression in certain pathological contexts. Furthermore, due to the structural diversity and functional complexity of the targets themselves, as well as the limited specificity and *in vivo* stability of current inhibitors, off-target toxicity remains a significant challenge. For instance, MCC950 was evaluated in a Phase II clinical trial for rheumatoid arthritis but was subsequently discontinued due to elevated serum liver enzyme levels observed in patients, Although the precise mechanism of its hepatotoxicity is not fully understood, it may be related to both its molecular structure and the high dosage used in clinical settings (Mangan et al., 2018). Therefore, bioavailability and the long-term safety of these agents require further validation in large-scale clinical trials.

## 6 Conclusion

The HMGB1/NLRP3 pathways serve as critical modulators in the development and progression of systemic inflammatory disorders. As pivotal drivers of innate immune responses, these pathways bridge inflammation and disease pathogenesis by orchestrating cellular stress signals, inflammasome activation, and the subsequent release of pro-inflammatory cytokines. Evidence increasingly underscores the centrality of HMGB1 and NLRP3 in promoting chronic inflammation and multi-organ damage across a spectrum of diseases, including autoimmune disorders, trauma, and stress. Notably, although the synergistic activation between HMGB1 and NLRP3 may form a pro-inflammatory positive feedback loop that accelerates disease progression, their regulatory mechanisms exhibit distinct variability and complexity across different tissue environments and pathological conditions. In particular, the functional roles and interactions of HMGB1 and NLRP3 may vary significantly depending on the cell type and stimulation context, and the ensuing activation of the associated inflammatory signaling cascades also displays diverse characteristics. In this review, we primarily describe the role of HMGB1/NLRP3 in systemic diseases and do not explore the related targeted therapeutic mechanisms in detail. Future studies should aim to unravel the precise molecular mechanisms underlying their interplay, enabling the development of highly specific, pathway-targeted interventions. Such approaches hold promise for improving outcomes in patients suffering from inflammation-driven systemic diseases. In summary, advancing our understanding of the HMGB1/NLRP3 axis will not only elucidate the molecular underpinnings of systemic inflammation but also pave the way for innovative therapeutic strategies to address the unmet medical needs in chronic inflammatory conditions.
